# Green and Stabilized Nanogold in *Eucheuma* Seaweed for Reactive Azo Catalysis

**DOI:** 10.1002/open.202500613

**Published:** 2026-04-16

**Authors:** Hong Wan, Mohammad Jahidul Alam, Sania Habib, Huihong Liu, Sakil Mahmud

**Affiliations:** ^1^ School of Life Science Wuchang University of Technology Wuhan People's Republic of China; ^2^ Faculty of Science Department of Chemistry University of Chittagong Chittagong People's Republic of Bangladesh; ^3^ School of Chemistry and Chemical Engineering Wuhan Textile University Wuhan People's Republic of China; ^4^ Ivory V. Nelson Center for the Sciences Department of Chemistry and Physics Lincoln University Oxford Pennsylvania USA; ^5^ Department of Textile Engineering Faculty of Engineering Daffodil International University Dhaka People's Republic of Bangladesh

**Keywords:** biogenic layer, electron micrographs, physicochemical, regression value, wastewater treatment

## Abstract

Gold nanoparticles (AuNPs) possess unique properties, making them highly versatile for various applications, though their large‐scale synthesis remains challenging. This article reports the biosynthesis of AuNPs using processed *Eucheuma* seaweed (PES). A sharp absorption band verified the successful formation of AuNPs at 542 nm in the UV‐visible spectrum. Electron microscopy showed quasi‐spherical particles with an average diameter of 11.41 ± 4.88 nm and high crystallinity (d‐spacing = 0.230 nm). PES biomolecules stabilized the AuNPs, as reflected by a zeta potential of −22.1 mV and the presence of carbon, sodium, oxygen, and nitrogen in energy diffraction and elemental mapping analyses. The AuNPs demonstrated 94.3% catalytic reduction of reactive orange 1 dye, with a high regression coefficient (*R* ^2^ = 0.984), indicating excellent catalytic efficiency and suitability for dye effluent treatment.

## Graphical abstract

Gold nanoparticles (AuNPs) were synthesized through a green approach using processed *Eucheuma* seaweed as a reducing and stabilizing agent. The resulting AuNPs showed efficient catalytic performance for the reduction of reactive orange 1 in the presence of sodium borohydride, highlighting their potential for ecofriendly dye catalysis.

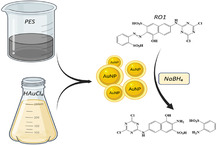

## 1 Introduction

Recent advancements in nanomaterials have been driven by their novel properties at the nanoscale, where surface and quantum phenomena dominate. These enhanced physicochemical characteristics, including mechanical strength, thermal stability, magnetism, electronic behavior, optical properties, and catalytic activity, confer significant advantages to nanoparticle‐based products [1, 2, 3, 4]. Metallic nanoparticles, in particular, exhibit unique absorption‐emission and photoluminescence properties due to localized surface plasmon resonance (LSPR), making them suitable for biomedical applications such as selective photothermal cancer treatment [5, 6, 7]. Furthermore, plasmonic nanometals can accelerate electron‐driven chemical reactions, enhancing reaction kinetics and boosting the efficiency of catalytic processes [8, 9]. The contamination of aquatic ecosystems with toxic pollutants, primarily from domestic and industrial waste, is a pressing environmental concern. These pollutants, originating from textile effluents, mining, sewage, wastewater, and agricultural runoff, include azo‐based colorants, which are widely used in the fabric industry for their stability and color range [10]. Azo dyes, characterized by the —N=N— bond, are also employed in industries like tanning, food processing, papermaking, cosmetics, and healthcare. However, a significant portion of these dyes remains in the effluent and is released into the environment, where they pose carcinogenic and mutagenic risks to both human health and marine life. When partially degraded, azo dyes generate toxic chemicals, making the decolorization of industrial effluents a critical step before environmental discharge [11, 12].

Various physicochemical techniques, including adsorption, membrane filtration, coagulation, flocculation, oxidation, and biological treatments, have been developed to mitigate the harmful effects of textile effluents. However, these methods often require secondary treatment to generate activated sludge, reducing their economic viability [13]. Metal nanoparticles offer a promising alternative for treating azo‐dye‐containing wastewater due to their enhanced surface reactivity, high catalytic properties, and ability to interact at the molecular level [14, 15]. Metal oxide nanoparticles, such as titanium dioxide [13], zinc oxide [16], and iron oxide [17], along with noble metal nanoparticles such as gold [18, 19] and silver [20, 21, 22] despite their promise, the large‐scale production of nanoparticles for wastewater treatment remains a challenge. The choice of synthesis method is crucial, as traditional techniques like sol–gel, coprecipitation, hydrothermal, solvothermal, and laser ablation require complex instrumentation and hazardous reagents [23, 24, 25] [26]. Moreover, surface modification of nanoparticles is necessary to address stability issues arising from their high surface area. Green synthesis offers an ecofriendly alternative for the sustainable production of nanoparticles, using benign materials and methods [27, 28]. Processed *Eucheuma* seaweed (PES), particularly κ‐carrageenan, is a naturally occurring polysaccharide widely recognized for its excellent binding, thickening, and stabilizing properties [29]. Beyond these functions, κ‐carrageenan exhibits intrinsic reducing and stabilizing capabilities, which have been extensively utilized in the green biosynthesis of metal nanoparticles [30, 31]. Recently, a limited number of polysaccharides have been studied for the green synthesis of gold nanoparticles (AuNPs) for dye catalysis (Table 1). However, the use of whole *Eucheuma* biomass, rather than an isolated polysaccharide, provides a complex multicomponent biological matrix comprising sulfated polysaccharides, proteins, and other bioactive constituents. These components act synergistically to regulate nanoparticle nucleation, growth, and surface functionalization; interactions that are inherently absent in single‐component polysaccharide systems, thereby offering a distinct nanomaterial design space.

**TABLE 1:** Summary of literature regarding the catalytic property of AuNPs.

Polysaccharides	Diameter, nm	Shape	Reaction types	Reference
Dextrin	8.0–28.0	Spherical	4‐NP reduction	[32]
Locust bean gum	—	Spherical	4‐NP reduction	[33]
Glucomannan	12.0–31.0	Spherical	4‐NP reduction	[34]
Katira gum	6.9	Spherical	4‐NP reduction	[35]
Starch‐g‐poly	11.1	—	4‐NP reduction	[36]
Dextrin	8.4–12.0	—	Liquid phase oxidation of ethylene glycol	[37]
Alginate	20.0–40.0	Centered cubic	Azo dyes	[38]
Water extract	8.2–12.8	Spherical	4‐NP reduction	[39]
Levan	5.0–12.0	Spherical	4‐NP reduction	[40]
Glucan	19.0–27.2	Spherical	4‐NP reduction	[41]

This table is adapted from Wang et al. [42] and reproduced under the CC BY 4.0 license.

In our previous work [43], κ‐carrageenan‐mediated AuNPs demonstrated effective catalytic reduction of reactive orange 7 and reactive yellow 145 dyes. Building on this foundation, the present study uses the entire PES to fabricate AuNPs via a single‐step, ecofriendly, extract‐free approach that avoids toxic chemicals. Compared to our earlier κ‐carrageenan‐mediated synthesis of AuNPs [43], the present study differs fundamentally in both material selection and conceptual scope. While purified κ‐carrageenan has previously been employed as a single‐component reducing and stabilizing agent, this work uses PES, a naturally derived multicomponent matrix rich in κ‐carrageenan and containing proteins, mineral ions, and other bioactive constituents. The presence of these additional components influences the reduction kinetics, nucleation behavior, and surface stabilization of AuNPs, enabling direct nanoparticle formation from minimally processed marine biomass. This approach eliminates the need for polysaccharide purification, offering a more sustainable and potentially scalable synthesis pathway, while providing new insight into the role of complex biogenic matrices in nanoparticle formation. Furthermore, comprehensive morphological characterization was conducted, and the catalytic efficiency of the biosynthesized AuNPs was evaluated for the previously unexplored reactive orange 1 (RO1) dye using sodium borohydride, demonstrating their potential for environmental remediation.

## 2 Experimental

### 2.1 Materials

κ‐Carrageenan‐enriched PES was obtained locally and used without additional purification. Chloroauric acid (HAuCl_4_), sodium borohydride (NaBH_4_), and sodium hydroxide (NaOH) of analytical grade were procured from Sinopharm Chemical Reagent Co., Ltd. (Shanghai, China). RO1 (CAS No. 6522‐74−3) was purchased from DyStar Colours Deutschland GmbH (Germany). All aqueous solutions were prepared using ultrapure water (18.3 MΩ·cm) produced by a Labpure Water System (Chengdu, China). Prior to use, all glassware was cleaned with a 3:1 (v/v) mixture of nitric acid (HNO_3_) and hydrochloric acid (HCl), thoroughly rinsed with distilled water, and air‐dried.

### 2.2 Green Synthesis of AuNPs

**SCHEME 1:**
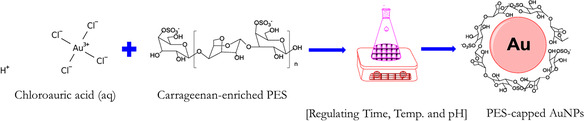
Complete synthesis procedure of AuNP by PES (Reproduced from Chen et al. [44] under the CC BY 4.0 license).

The synthesis of AuNPs followed the procedure outlined in our previous work [43]. To prepare the PES solution, 7 g of PES powder was added to 100 mL of distilled water and stirred at 65°C for 2 h to facilitate thorough dissolution. The mixture was left to stand overnight at room temperature and then filtered. For the synthesis of AuNPs, a 0.4 mM chloroauric acid (HAuCl_4_) solution was combined with 0.30 mL of PES solution and heated at 80°C for 30 min in a slightly acidic environment (pH 6). The formation and morphological characteristics of AuNPs were investigated using various analytical techniques. A schematic diagram of the complete AuNP synthesis process is shown in Scheme 1.

### 2.3 Characterization and Measurement

Morphological analysis of the synthesized nanomaterials was conducted using a scanning electron microscope (SEM, JEOL 6460LV, Tokyo, Japan) equipped with an Oxford Instruments energy‐dispersive X‐ray spectroscopy (EDX) detector and Inca automated mapping software for quantitative analysis. The detailed nanostructure and composition were further examined by high‐resolution transmission electron microscopy (HRTEM, JEOL JEM‐2100F, Japan).

The catalytic efficiency of the synthesized AuNPs for the reduction of RO1 azo dye was assessed spectroscopically, following the method described by Lin et al. [45]. In brief, the catalytic activity of AuNPs toward azo‐dye reduction was assessed by adding 100 μL of NaBH_4_ (0.2 M) to 3 mL of dye solution (30 mg/L) in a 10 mL flask, followed by 30 μL of AuNPs suspension. The mixture was rapidly stirred and immediately transferred to a quartz cuvette for analysis. The reaction progress was monitored using UV–Visible spectroscopy (TU‐1901, Purkinje General Instrument Co. Ltd., Beijing, China) over the range of 200–700 nm with a 1 cm path‐length quartz cell. The catalytic efficiency was determined based on the temporal decrease in dye absorbance.

## 3 Result and Discussion

### 3.1 Synthesis and Characterization of AuNPs

**SCHEME 2:**
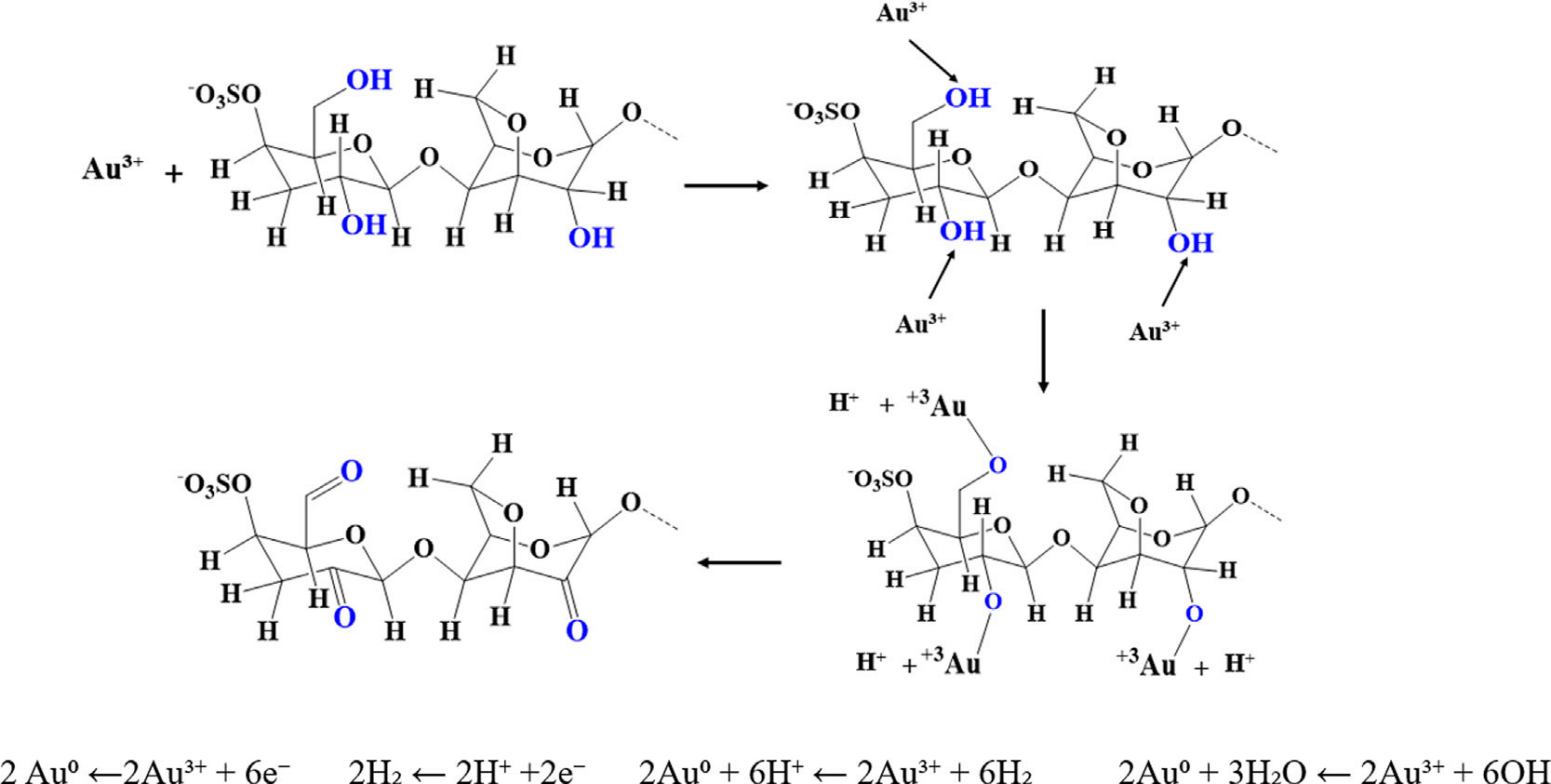
Formation mechanism of AuNPs.

Upon mixing the yellow HAuCl_4_ solution with PES, the gray hue of the PES solution initially transitioned to a pale pink. Under optimized synthesis conditions, the mixture gradually turned purplish‐red, indicative of LSPR, signaling the successful formation of AuNPs. The LSPR effect arises when the electric field of incident light induces electronic oscillations in AuNPs, creating a dipole moment and thereby enhancing the electric field near the particle surface, thereby amplifying the LSPR response [46]. As demonstrated in our previous work [43], UV–visible absorption spectra of AuNPs, HAuCl_4_, and PES solutions further confirmed the bioreduction of Au^3+^ to AuNPs. A characteristic absorption peak at 304 nm, attributed to the LSPR of Au^3+^ ions in the HAuCl_4_ spectrum, disappeared during the reaction. In its place, a new absorption band at 542 nm, associated with AuNPs, confirmed the successful synthesis of AuNPs [47]. The role of PES in both the fabrication and stabilization of the AuNPs was crucial, as depicted in Scheme 2. It is worth noting that, compared to pure κ‐carrageenan [43], the PES‐mediated synthesis presented here operates in a chemically heterogeneous biogenic environment. Unlike purified κ‐carrageenan, PES contains multiple functional groups and inorganic ions that jointly contribute to AuNP reduction and stabilization, influencing particle size, surface charge, and catalytic behavior.

Initially, the reduction of Au^3+^ ions from tetrachloroaurate ions (AuCl_4_^−^) is facilitated by the polysaccharides present in PES, which serve as reducing agents [48]. In the early stages, the growth rate of AuNPs is slow as the Au^3+^ ions become activated. However, as the ionic interactions between Au^3+^ ions and PES biomolecules intensify, the reduction process accelerates, leading to the nucleation of AuNPs. The extent of nucleation determines the morphology of the nanoparticles. Biomolecules from PES adsorb onto AuNP surfaces via ionic interactions between surface charges and their π‐conjugated systems, stabilizing the particles. As the reduction proceeds, additional Au^3+^ ions aggregate around the nuclei, promoting the growth and agglomeration of AuNPs. The overall mechanism of AuNP formation and stabilization mediated by PES is outlined in Equation (1)–(6).

(1) $${\text{HAuCl}}_{4\left(\mathrm{aq}\right)}\;\to \;H_{\left(\mathrm{aq}\right)}^{+}+{\text{AuCl}}_{4\left(\mathrm{aq}\right)}^{-}$$

(2) $${\text{AuCl}}_{4\left(\mathrm{aq}\right)}^{-}+\mathrm{PES}\;\to \;{\mathrm{Au}}_{\left(\mathrm{aq}\right)}^{0}+\;4{\mathrm{Cl}}_{\left(\mathrm{aq}\right)}^{-}+\text{Oxidized PES}$$

(3) $${\mathrm{nAu}}^{0}\;\to \;{\left(\mathrm{Au}\right)}_{n}$$

(4) $$\begin{array}{l} {\left(\mathrm{Au}\right)}_{n}+{\mathrm{Au}}_{\left(\mathrm{aq}\right)}^{3+}+\text{Oxidized PES}\;\to \;{\left(\mathrm{Au}\right)}_{n+1}\;\left(\text{nuclei}\right)\\ \;+\left[\text{Reduced PES}\right] \end{array}$$

(5) AuNPs+PES → Stable AuNPs‐PES Complex

(6) $${\mathrm{Au}}^{3+}+\mathrm{PES}\;\to \;\text{Stable AuNPs}+\text{by‐products}$$

**FIGURE 1:**
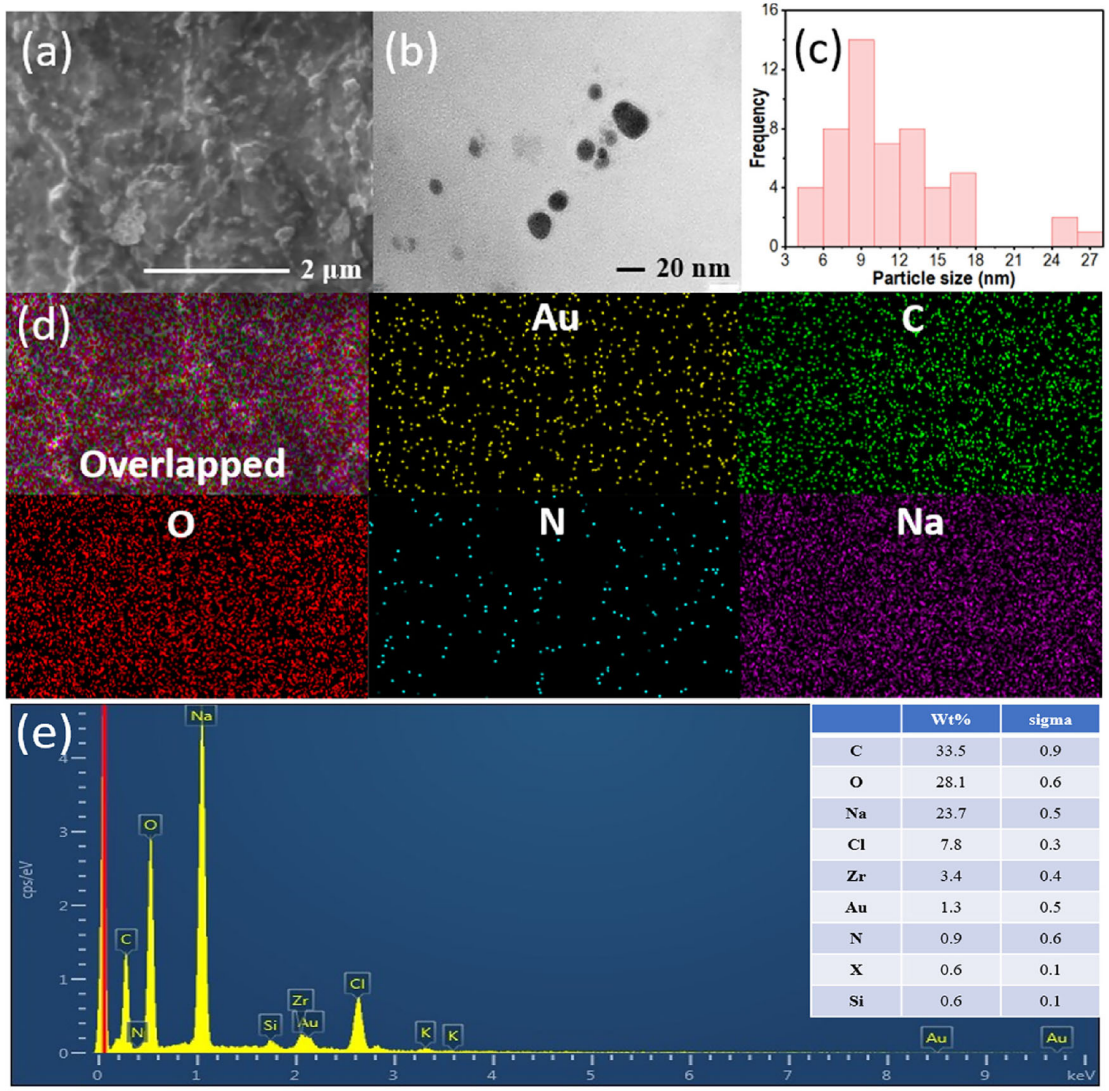
(a) SEM image displaying the surface morphology, (b) TEM image with a nanoscale measurement bar, (c) histogram showing particle size distribution, (d) elemental mapping obtained via SEM, and (e) EDX spectrum with elemental composition (wt%) listed in the inset, all for AuNPs synthesized using PES at optimized synthesis conditions.

While previous methods have been employed to synthesize AuNPs [43], the current batch of samples was comprehensively characterized to confirm successful synthesis. The morphological and topographical characteristics of AuNPs are critical for their diverse applications [49, 50]. To investigate these properties, we initially employed SEM, which revealed AuNPs of varying sizes and shapes embedded within the PES matrix (Figure 1a). However, the SEM offered a resolution of approximately 2 µm, confining the analysis to the micrometer scale and limiting detailed assessment of nanoscale morphology [51]. To overcome this, we used TEM, which revealed quasi‐spherical AuNPs, well‐separated and uniformly distributed over the PES matrix (Figure 1b). The particle size distribution histogram indicated an average diameter of 11.412 ± 4.88 nm, confirming successful nanoscale fabrication (Figure 1c).

HRTEM images further revealed well‐defined crystalline surfaces, evidenced by distinct lattice fringes with a d‐spacing of 0.230 nm, corresponding to the face‐centered cubic structure of Au, representing the (111) and (200) planes. The zeta potential of −22.7 mV confirmed the presence of a PES‐mediated stern layer on the AuNPs surface, which imparted a negative charge to the biogenic layer. This negative charge generated a strong repulsive force, preventing nanoparticle aggregation and stabilizing the colloidal solution [52]. Additionally, PES acted as a capping agent, regulating the formation of predominantly spherical AuNPs. This result aligns with Daizy's findings on the role of biomolecules in honey in shaping AuNP morphology [53]. SEM‐derived elemental mapping showed extensive color contrast, indicating uniform AuNP dispersion over the surface (Figure 1d). The presence of oxygen (O) and carbon (C) elements, associated with the PES macromolecule, further confirmed PES's role as a capping agent [52]. The elemental composition was examined by energy‐dispersive X‐ray spectroscopy (EDX) (Figure 1e). Characteristic peaks corresponding to Au were observed, confirming the presence of gold in the composite, with a weight percentage of 1.3% (Figure 1e, inset). In addition, signals from Na, O, and C were detected, originating from residual NaOH and the PES matrix, respectively. The EDX results are consistent with the elemental mapping analysis, indicating successful incorporation and uniform distribution of Au within the composite. Parallel findings were observed by Sunday et al. [54] during the biosynthesis of AuNPs using *Garcinia kola* pulp extract.

### 3.2 Catalysis of Reactive Azo Dye

**FIGURE 2:**
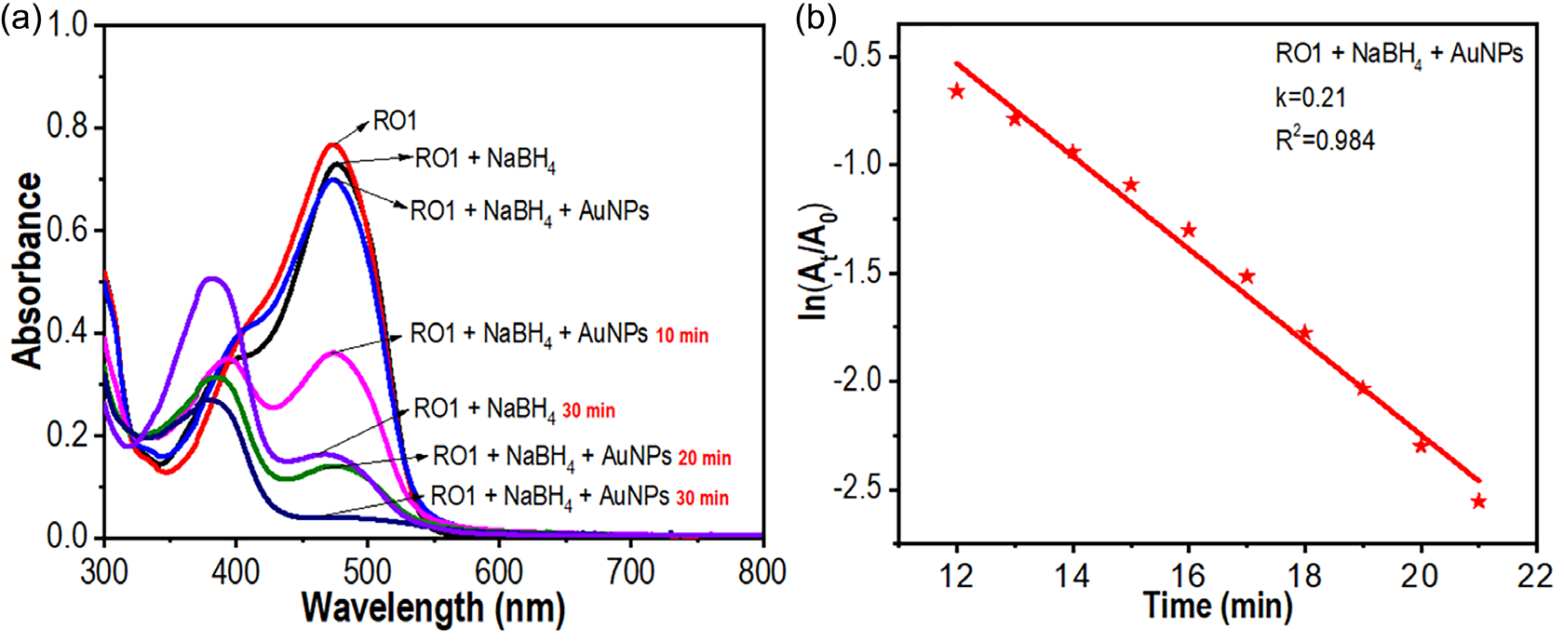
(a) UV–Vis spectrum and (b) kinetic diagram of RO1 treated with AuNPs and NaBH_4_.

The delocalized π‐electrons in the conjugated system of azo dyes absorb energy from visible light, undergoing π–π* electronic transitions. This absorption results in the reflection of complementary colors, which gives the dye its characteristic hue [55, 56]. To investigate the catalytic efficiency of AuNPs for the catalytic reduction of RO1, spectroscopic studies were conducted in the presence of sodium borohydride (NaBH_4_), a reducing agent. The catalytic reduction percentage (*D*%) was calculated using the formula: [*D*% = (*A*_0_ – *A*)/ *A*_0_ × 100], where *A*_0_ indicates the initial absorbance, and *A*_t_ is that of at a specific time (*t*) [57]. Initially, treating RO1 with NaBH_4_ alone resulted in a modest reduction, as evidenced by a slight decline in the intensity of the absorption peak at 473 nm in the UV–Vis spectrum (Figure 2a). This yielded a catalytic reduction percentage of 76.9% after 30 min, indicating partial cleavage of the azo bonds. However, the addition of AuNPs alongside NaBH_4_ led to a near‐complete disappearance of the RO1 peak within just 20 min. The corresponding color change, as the dye solution faded, signified successful reduction. The catalytic reduction percentage increased to 94.3%, demonstrating the superior catalytic efficacy of AuNPs as nanocatalysts for azo dye reduction. Given the excess concentration of NaBH_4_ compared to RO1, the reaction kinetics were treated as pseudo‐first order [58]. A plot of −ln (*A*_t_/*A*_0_) versus time displayed a linear correlation, with a regression coefficient (*R* ^2^) of 0.984, affirming the reaction's kinetic behavior (Figure 2b). A comparative summary of the photocatalytic reduction efficiencies of AuNPs synthesized using various natural compounds for different azo dyes is provided in Table 2.

**TABLE 2:** Reduction of various azo dyes with AuNPs in the presence of NaBH_4_.

Nanoparticles	Particle size, nm	Dyes	***K*, min** ^**−1**^	*R* ^2^	*D* %	Reference
AuNPs‐PES	11.412 ± 4.88	RO1	0.21	0.984	94.3	This work
AuNPs‐BM	∼14	CR	0.2192	—	98	[57]
AuNPs‐LI	20 ± 13.0	MR	0.013	0.98	91	[59]
AuNPs‐Asp.	39.7	MO	0.314	0.972	95	[60]
AuNPs‐Asp.	39.7	AR27	0.228	0.997	98	[60]
AuNPs‐DLP	∼10.5	CR	4.5 × 10^−3^	0.9959	—	[61]
AuNPs‐DLP	∼10.5	MO	1.7 × 10^−3^	0.9918	—	[61]

Abbreviations: AR27 Acid red 27; Asp., L‐asparagine; BM, B. marisflavi; CR, Congo red; *D*%, Degradation rate; DLP, Dalspinin; LI, Lawsoniainermis; MO, Methyl orange; MR, Methyl red; RO1, Reactive orange 1.

**SCHEME 3:**
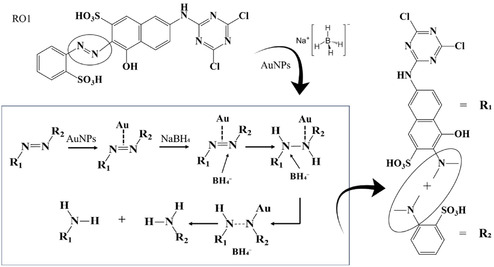
Catalytic reduction of Reactive Orange 1 with AuNPs in the presence of NaBH_4_.

NaBH_4_ functions as a potent reducing agent, releasing hydride ions (H^−^) in aqueous solution that act as electron donors for the reduction of RO1. However, the reduction of RO1 by NaBH_4_ alone is kinetically sluggish due to the high activation energy barrier. AuNPs play a critical catalytic role in overcoming this limitation by providing high‐surface‐area active sites that facilitate efficient electron transfer [62]. By adsorbing both ${\mathrm{BH}}_{4}^{‐}$ and RO1 onto their surfaces, AuNPs enhance the reduction process, accelerating electron flow from hydride ions to the azo dye, thereby expediting the catalytic reduction of RO1. The catalytic reduction of RO1 in the presence of NaBH_4_ and AuNPs proceeds through a multistep mechanism [63]. Initially, both RO1 and ${\mathrm{BH}}_{4}^{‐}$ ions adsorb onto the surface of AuNPs. The ${\mathrm{BH}}_{4}^{‐}$ ions act as a source of hydride ions (H^−^), while the RO1 molecules interact with the nanoparticle surface via electrostatic or Van der Waals forces. The AuNPs serve as highly efficient electron transfer mediators, significantly lowering the activation energy required for the reduction of the azo bond (—N=N—) in RO1. This facilitates the transfer of hydride ions from ${\mathrm{BH}}_{4}^{‐}$ to the azo bond, reducing it to two amine groups (—NH_2_). The reduction disrupts the dye's chromophore, resulting in decolorization. After reduction, the resulting aromatic amine products desorb from the AuNPs surface, allowing the nanoparticles to be regenerated for additional catalytic cycles [64]. The overall reaction steps involved in the catalytic reduction of RO1 by AuNPs and NaBH_4_ are summarized in Equation (7)–(15) and Scheme 3.

(7) $$\begin{array}{c} R[chemistry single bond solid line]N[chemistry double bond solid lines]N[chemistry single bond solid line]R^{\prime }+\text{AuNPs}\;(\text{surface})\\ \;\to \;R[chemistry single bond solid line]N[chemistry double bond solid lines]N[chemistry single bond solid line]R^{\prime }\;\left(\text{adsorbed}\right) \end{array}$$

(8) $${\text{NaBH}}_{4}+\text{AuNPs }\left(\text{surface}\right)\;\to \;{\text{NaBH}}_{4}\;\left(\text{adsorbed}\right)$$

(9) $${\text{NaBH}}_{4}\;\left(\text{adsorbed}\right)\;\to \;\text{AuNPs}+{\mathrm{BH}}_{4}^{-}+\text{electron relay}$$

(10) $$\begin{array}{l} R[chemistry single bond solid line]N[chemistry double bond solid lines]N[chemistry single bond solid line]R^{\prime }\left(\text{adsorbed}\right)+\text{electron relay}\\ \;+\text{AuNPs}\;\to \;R[chemistry single bond solid line]\mathrm{HN}[chemistry single bond solid line]\mathrm{NH}[chemistry single bond solid line]R^{\prime }\;\left[\text{Color fading}\right] \end{array}$$

(11) $$\begin{array}{l} R[chemistry single bond solid line]\mathrm{HN}[chemistry single bond solid line]\mathrm{NH}[chemistry single bond solid line]R^{\prime }+H_{2}\;\to \;R[chemistry single bond solid line]{\mathrm{NH}}_{2}\\ \;+R^{\prime }[chemistry single bond solid line]{\mathrm{NH}}_{2}\;\left[\text{Complete decolorization}\right] \end{array}$$

(12) $$\begin{array}{l} {\left[R[chemistry single bond solid line]{\mathrm{NH}}_{2}\right]}_{\left(\mathrm{ads}\right)}+{\left[R^{\prime }[chemistry single bond solid line]{\mathrm{NH}}_{2}\right]}_{\left(\mathrm{ads}\right)}\;\to \;R[chemistry single bond solid line]{\mathrm{NH}}_{2}+R^{\prime }[chemistry single bond solid line]{\mathrm{NH}}_{2}\\ \;+\text{AuNPs }\left[\text{Product desorption and catalyst regeneration}\right] \end{array}$$

(13) $$\begin{array}{l} R[chemistry single bond solid line]N[chemistry double bond solid lines]N[chemistry single bond solid line]R^{\prime }+4{\text{NaBH}}_{4}+2H_{2}O\;\to \;R[chemistry single bond solid line]{\mathrm{NH}}_{2}+R^{\prime }[chemistry single bond solid line]{\mathrm{NH}}_{2}\\ \;+4{\text{NaBO}}_{2}\;\left[\text{Combined reaction}\right] \end{array}$$

(14) $$\begin{array}{l} \text{AuNPs}+{\mathrm{BH}}_{4}^{-}\;\to \;\text{AuNP }\left(e^{-}\right)+\text{Oxidation Products}\\ \;[\text{Overall catalytic cycle concept}] \end{array}$$

(15) $$\begin{array}{l} \text{AuNPs }\left(e^{-}\right)+\mathrm{Dye}\;\to \;\text{AuNP}+\text{Reduced Products}\\ \;[\text{Overall catalytic cycle concept}] \end{array}$$

The recovery and reuse of AuNP catalysts are vital for their practical and industrial application, as well as environmental concerns. As indicated by Equation (12), AuNPs are regenerated during the step‐by‐step reduction of RO1, suggesting their potential for repeated catalytic cycles. However, separation of AuNPs from aqueous colloidal media after synthesis, or from reaction solutions following dye degradation, remains challenging, which can limit direct reuse. Although catalyst recyclability was not evaluated in the present study, our previous work has demonstrated effective strategies to overcome this limitation. For example, colloidal AuNPs were converted into solid spherical beads via controlled gelation in CaCl_2_ solution, followed by stabilization and drying, making them suitable for reuse [38, 65]. Additionally, immobilization or incorporation of nanocatalysts onto solid substrates such as fibers or membranes has been shown to facilitate straightforward recovery and repeated catalytic operation [66, 67]. Building on these approaches, future studies will focus on detailed characterization of post‐reaction solutions and the development of recyclable catalytic platforms to enhance the practical applicability of the system.

## 4 Conclusion

In summary, the single‐step bioreduction method employed in this study adhered to green synthesis principles, effectively reducing Au^3+^ ions to AuNPs in a colloidal solution using only the polysaccharide‐rich PES, without the need for hazardous chemicals. PES also played an essential role in stabilizing the AuNPs by forming a biogenic layer around the particles, preventing aggregation. Morphological and topographical analyses confirmed the successful formation of predominantly spherical, well‐separated, and uniformly dispersed AuNPs, with a narrow size distribution of 11.412 ± 4.88 nm. Moreover, the catalytic application of these AuNPs for dye catalysis demonstrated their potential as an efficient alternative for reduction reactions, achieving a high catalytic reduction efficiency (*D*% = 94.3%). The AuNPs not only facilitated the reduction process by lowering the activation energy but also accelerated the reaction kinetics. This green synthesis method shows great promise for scalable industrial production of AuNPs, offering a sustainable alternative for traditional wastewater treatment technologies. Future work will specifically address catalyst recovery, multicycle reuse, and post‐reaction morphological stability through immobilization or support‐based strategies, while preserving the green and single‐step advantages of the *Eucheuma*‐mediated synthesis.

## Disclosure and Declaration of AI Use

Generative AI tools were used solely to enhance language and readability. All content was subsequently reviewed and edited by the authors, who take full responsibility for the final version.

## Funding

This study was supported by Key Laboratory of Biomass Fibers (grant STRZ2020001), Eco‐Dyeing and Finishing (grant STRZ2020011). This work was also supported by the Cross‐Cutting Project of Wuchang University of Technology (HZ2021036) and the Scientific Research Program of Wuchang University of Technology, China (X2024ZZ004).

## Conflicts of Interest

The authors declare no conflicts of interest.

## Data Availability

The data that support the findings of this study are available from the corresponding author upon reasonable request.
